# Let's Talk about Moving: The Impact of Cardiorespiratory Fitness, Exercise, Steps and Sitting on Cardiovascular Risk

**DOI:** 10.21470/1678-9741-2016-0078

**Published:** 2017

**Authors:** Ross Arena, Amy McNeil

**Affiliations:** 1 Department of Physical Therapy, Department of Kinesiology and Nutrition, College of Applied Health Sciences, University of Illinois at Chicago, Chicago, Illinois, USA.

A preferred treatment for cardiovascular disease (CVD) is preventing its occurrence (*i.e.*, primary prevention). Moving further upstream, preventing the occurrence of well-established CVD risk factors from ever manifesting is optimal (*i.e.*, primordial prevention). In the unfortunate and currently common situation where CVD does manifest, either clinical or subclinical, reversal of risk factors becomes imperative (*i.e.*, secondary prevention). Regardless of the CVD prevention entry point (*i.e.*, primordial to secondary), increasing physical activity and cardiorespiratory fitness (CRF) is a primary objective^[1-3]^.

While unique entities, there is a degree of overlap between physical activity and CRF that warrants recognition. Physical activity is quantified by some type of activity tracker or more commonly by self-report. CRF is quantified by exercise testing techniques, where peak/maximal aerobic capacity is either estimated [*i.e.*, metabolic equivalents (METs)] or directly measured [*i.e.*, peak oxygen consumption (VO_2_)]. The link between both higher levels of physical activity and CRF and a decreased risk of being diagnosed with CVD, or suffering a subsequent adverse event if already diagnosed with CVD, is beyond dispute^[1,2,4,5]^. Comparatively, the drop in CVD risk with increasing CRF is sharper than that observed with increasing levels of physical activity, an observation likely associated with the fact that the former is a more objective measure that the latter.

There are well established recommendations for weekly exercise patterns: 1) 150 minutes or more moderate-intensity aerobic activity per week; or 2) 75 minutes or more of vigorous aerobic activity per week^[6]^. Participating in exercise at these volumes portends clear health benefits^[7,8]^. Even so, it has become increasingly recognized that exercise volumes falling significantly below these recommendations also provides substantial health benefits. For example, Lavie et al.^[3]^ recently reviewed the risk of cardiovascular and all-cause mortality according to running behaviors (*i.e.*, minutes, distance and frequency per week) divided into quintiles. Compared to non-runners, the greatest risk reduction was realized in the first quintile, which equated to running <51 minutes per week, less than 6 miles per week, and at frequency of 1-2 times per week. In over 3,000 individuals ≥65 years, Sundquist et al.^[9]^ found those who reported exercising once a week had a 40% lower all-cause mortality risk compared to those who reported no weekly exercise. This volume of exercise is well below the current recommendations and yet elicits significant risk reduction.

Improvements in CRF follows a similar incremental pattern with respect to reductions in CVD risk; each 1 MET improvement in CRF, particularly between the 5-10 peak MET level^[10]^, equates to a 10-30% risk reduction for premature mortality^[2]^. In a large meta-analysis of healthy men and women, Kodama et al.^[11]^ found all-cause mortality and CVD risk decreased 13% and 15%, respectively, per 1 MET increase in CRF. In a larger cohort of patients with CVD undergoing CR, Martin et al.^[12]^ reported a 13% reduction in mortality per 1-MET increase in patients with a high baseline CRF level (*i.e.*, >8 METs) and a 30% reduction in mortality per 1-MET increase in patients with a low baseline CRF level (*i.e.*, <5 METs). There is a dearth of additional investigations demonstrating a similar trend. These consistent findings highlight that modest improvements in CRF, commonly not to a level that would allow for the attainment of a normal age/sex predicted level, can lead to significant health benefits. The strength by which CRF level predicts future health trajectory has prompted discussion of this measure being consider a vital sign^[10,13]^.

Time spent participating in a structured exercise program and CRF are often the exclusive measures used to gauge an individual's risk for CVD. There is data to suggest value in expanding measures to assess a more comprehensive "movement portfolio" when determining CVD risk and providing guidance to improving one's health trajectory through a broader array of activities that require physical exertion.

Achieving 10,000 steps per day is now a commonly cited goal, although not considered in the context of a structured exercise program^[14,15]^. Those who take more steps per day clearly have better health outcomes^[14-16]^ and the relationship between step count and health benefits follows a linear, continual scale. For example, Dwyer et al.^[16]^ found each 1,000 daily step count increase resulted in a 6% reduction in all-cause mortality.

The health detriments of prolonged sedentary time is also becoming increasingly clear^[17]^. This is a significant health concern given adults average 6-8 hours of sedentary time each day and increases with advancing age^[18]^. Pandey et al.^[19]^ conducted a large meta-analysis (*i.e.* 700,000 subjects) and found a nonlinear significant increase in risk for poor health outcome when sedentary time exceeded 10 hours per day. Chau et al.^[17]^ found a similar association; ≥10 hours of total sitting time equated to a 65% higher risk of all-cause mortality compared to subjects who sat <4 hours per day. A recent American Heart Association scientific advisory on "Sedentary Behavior and Cardiovascular Morbidity and Mortality" highlighted the importance of decreasing sedentary time and concluded by stating "sit less, move more"^[18]^.

Given the apparent importance of an expanded movement portfolio, perhaps clinicians should consider incorporating "movement as a vital sign" and ask the following questions: 1) How many steps do you take each day?; 2) How many hours do you spend sitting each day?; 3) Do you interrupt sitting time with movement and if so what type and how often?; 4) Do you participate in a regular exercise program and if so how many times per week, what intensity, how long is each session and what type of exercise do you perform?; and 5) If you have had an exercise test, what is your CRF? Embracing a "Movement as a Vital Sign" framework and asking these five questions potentially allows for an orchestrated dialogue, between the clinician and patient, on how to progressively increase movement patterns using a pragmatic, individualized approach, which may be viewed as substantially more achievable by the patient.

In conclusion, moving more portends a clear health benefit with respect to reducing CVD risk across the prevention spectrum (*i.e.*, primordial to secondary). Moreover, current evidence indicates there is a need to reconsider how we counsel individuals on becoming more physically active, allowing for a more pragmatic and achievable approach. As healthcare providers, we should all be prepared to "talk about moving more" with those we are charged with caring for during every encounter.
